# A lightweight fire detection algorithm for small targets based on YOLOv5s

**DOI:** 10.1038/s41598-024-64934-4

**Published:** 2024-06-19

**Authors:** Changzhi Lv, Haiyong Zhou, Yu Chen, Di Fan, Fangyi Di

**Affiliations:** 1 National Experimental Teaching Demonstration Center for Electrical Engineering and Electronics, College of Electrical Engineering and Automation, Shandong University of Science and Technology, Qingdao, 266590 Shandong China; 2 College of Electrical Engineering and Automation, Shandong University of Science and Technology, Qingdao, Shandong China; 3 College of Electronic Information Engineering, Shandong University of Science and Technology, Qingdao, Shandong China

**Keywords:** Engineering, Materials science, Mathematics and computing

## Abstract

In response to the current challenges fire detection algorithms encounter, including low detection accuracy and limited recognition rates for small fire targets in complex environments, we present a lightweight fire detection algorithm based on an improved YOLOv5s. The introduction of the CoT (Contextual Transformer) structure into the backbone neural network, along with the creation of the novel CSP1_CoT (Cross stage partial 1_contextual transformer) module, has effectively reduced the model’s parameter count while simultaneously enhancing the feature extraction and fusion capabilities of the backbone network; The network’s Neck architecture has been extended by introducing a dedicated detection layer tailored for small targets and incorporating the SE (Squeeze-and-Excitation) attention mechanism. This augmentation, while minimizing parameter proliferation, has significantly bolstered the interaction of multi-feature information, resulting in an enhanced small target detection capability; The substitution of the original loss function with the Focal-EIoU (Focal-Efficient IoU) loss function has yielded a further improvement in the model’s convergence speed and precision; The experimental results indicate that the modified model achieves an mAP@.5 of 96% and an accuracy of 94.8%, marking improvements of 8.8% and 8.9%, respectively, over the original model. Furthermore, the model’s parameter count has been reduced by 1.1%, resulting in a compact model size of only 14.6MB. Additionally, the detection speed has reached 85 FPS (Frames Per Second), thus satisfying real-time detection requirements. This enhancement in precision and accuracy, while simultaneously meeting real-time and lightweight constraints, effectively caters to the demands of fire detection.

## Introduction

Traditional fire detection technology relies mainly on sensors, of which temperature and smoke sensors are the most widely used sensors, which convert temperature and smoke information into electrical signals with high accuracy and sensitivity^1^. With the development of sensor technology, infrared fire sensors can detect the occurrence of a fire by taking advantage of the fact that the burning of a substance produces a specific spectrum of radiation as it is not dependent on the temperature or smoke produced by the fire^2^. However, this method also has the disadvantage of being susceptible to interference from other sources of radiation. In recent years, Kort et al. detected carbon dioxide levels in the environment by means of carbon dioxide sensors and sediment quality for fire early warning^3^, but the sediment quality detection equipment may require regular cleaning and maintenance, which increases maintenance costs. Liu et al. achieved fire detection by deploying a network of fibre-optic temperature sensors to monitor temperature profiles in real time^4^, which allows for more comprehensive temperature detection but is not applicable to complex outdoor situations.

In order to address the challenges faced by traditional sensor technologies in dealing with complex fire scenarios, image processing techniques have been applied to fire detection, enabling the timely detection of fires through the analysis and processing of image or video data^5,6^. However, traditional image processing techniques rely on the manual extraction of fire-related features, such as the colour and texture of fires^7,8^. In reality, fire incidents can vary significantly in terms of type and scene complexity, and there are numerous uncertainties involved. Image processing algorithms.

that depend on the manual extraction of fire features lack strong generalization capabilities, making them susceptible to issues such as false positives and false negatives^9^.

In recent years, image-based fire detection technology has emerged as a prominent research focus, with significant advancements in deep learning being made in the field of object detection^10^. Compared to traditional methods, deep learning-based fire detection approaches leverage the capability of deep neural networks to extract more abstract and hierarchical features from images^11^. As a result, deep learning models exhibit stronger generalization abilities^12^. In 2016, Frizzi et al. made a pioneering contribution by employing convolutional neural networks to automatically learn and extract complex image features from fire and smoke images, enabling the detection of fire and smoke^13^. In 2020, Li et al. conducted a comparative study of various object detection models, including Faster-RCNN, R-FCN, SSD, and YOLOv3, in the context of fire detection^14^. Their findings ultimately concluded that the RCNN series of models, with larger models, longer training time and slower speed of detection but the YOLOv3 model demonstrated superior performance in terms of both accuracy and speed of fire detection. In 2020, Yue et al. integrated the K-Means +  + clustering algorithm into the YOLOv3 model to obtain suitable anchor boxes, resulting in improved accuracy for fire detection compared to the original model^15^. In 2021, Avazov et al. achieved a reduction in model parameters by modifying the YOLOv4 network while maintaining its detection accuracy. They further deployed this optimized model onto a BPI M3 development board, enabling the device to trigger an alarm within 8 s of a fire incident^16^. In 2022, a Bi-FPN (Bidirectional Feature Pyramid Network) was introduced in PANet (Path Aggregation Network), a feature fusion network for YOLOv4, which effectively reduced false positives in fire detection^17^. Additionally, they incorporated a ViBe threshold adaptive mechanism to enable dynamic detection of fires. In 2022, An et al. integrated dynamic convolution into the YOLOv5 model^18^. They also performed pruning and optimization on the YOLOv5 network, achieving an enhancement in the precision and accuracy of fire detection while reducing computational costs. In 2023, Ju et al. introduced the CBAM (Convolutional Block Attention Module) attention mechanism and depth-wise separable convolution into the YOLOvX-tiny model^19^. This led to a reduction in the network model parameters while improving the precision of object detection. Additionally, the optimized model can be deployed on embedded devices, further expanding its practical applicability. In 2023, Song et al. enhanced the target detection accuracy of the YOLOv5s model in the fire detection task by introducing a bidirectional cross-scale fusion module and a coordinated attention mechanism. And the generalisation of the model was further enhanced by adjusting and expanding the dataset using various data enhancement techniques^20^. In 2024, Geng et al. proposed a high-precision smoke detection algorithm named YOLOFM^21^, which improves the key indexes such as accuracy and mAP@0.5, but may be disturbed when facing some complex detection environments.

In summary, the two-stage model represented by the R-CNN^22^ series cannot meet the requirement of real-time fire detection^23,24^. The single-stage model represented by the YOLO series^25–27^ can better balance the accuracy and speed of fire detection, but in the early stage of a fire, the fire is mostly a small target and is often accompanied by unfavourable factors such as light transformation and background interference, which leads to a serious problem of fire misdetection and leakage rate, so the YOLO model can not be directly used for fire detection. To address the aforementioned challenges, we improved YOLOv5s based on the lightweight version of YOLOv5.

The main contributions of our work are as follows:

1. In order to further reduce the number of parameters of the improved YOLOv5s network and improve the feature extraction ability of fires, this paper introduces the CoT network into the CSP1 module, creates a CSP1_CoT structure, and replaces the original CSP1 module in the backbone network.
2. In order to strengthen the ability of the improved YOLOv5s network to fuse the features of fire, this paper combines the SE attention mechanism with the CSP2 module to create a CSP2_SE (Cross Stage Partial 2-Squeeze-and-Excitation) module to replace the original CSP2 module in the neck network.
3. In order to enhance the detection capability of small target fires, a special fire detection layer for small targets was added, and the YOLOv5s network was changed from the original three-scale detection to four-scale detection.
4. The loss function of the YOLOv5s network adopts CIoU, and the CIoU loss function does not consider the imbalance of difficult and easy samples, so the loss function of the model is changed to Focal-EIoU, which further improves the accuracy of the model and accelerates the convergence of the model. These collective improvements have resulted in a model that balances lightweight design with real-time performance while improving the accuracy and precision of fire detection.

The rest of this article is organized as follows. The “Methodology” section provides a comprehensive introduction to YOLOv5s, CSP1_CoT, CSP2_SE, Small object detection layer, Focal-EIoU, and improved fire detection algorithms. The “Experiment and results” section gives the results and some detailed discussions. The “Conclusions” section summarizes the work of this article.

## Methodology

### YOLOv5s algorithm

YOLOv5s is the smallest single-stage object detection model in the YOLOv5 series, optimized for computational efficiency while maintaining high detection accuracy. Therefore, we construct the fire detection model based on the YOLOv5s network.

**Figure 1:**
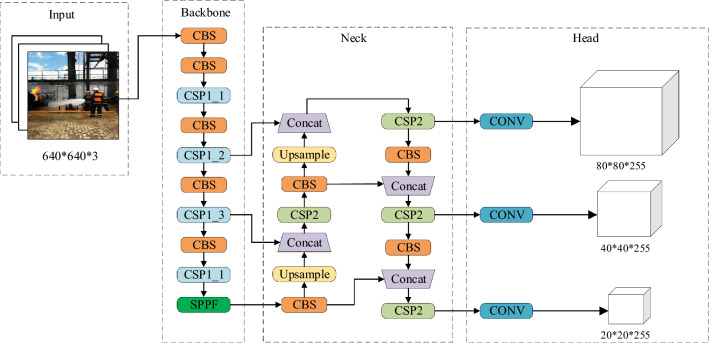
YOLOv5s network structure.

The architecture of the YOLOv5s network is illustrated in Fig. 1. The YOLOv5s network is composed of several key components, including the Input layer, the Backbone consisting of CBS, CSP1_X, and SPPF (Spatial Pyramid Pooling Fast) modules, the Neck composed of CSP2, Upsample, and Concat modules, and three distinct-scale prediction heads (Head).

**Figure 2:**
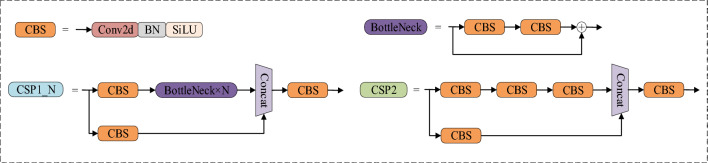
CSP structure.

The CSP module is the key module of YOLOv5s to improve the detection performance, which is composed of several specially designed CBS submodules^28^, each CBS module contains a convolutional layer, a BN (Batch Normalization) layer and a SiLU (Sigmoid Linear Unit) activation layer. Different numbers of CBS submodules constitute two types of CSP modules, namely CSP1_N module and CSP2 module. The structure diagram is shown in Fig. 2. The CSP1_N module is introduced in the Backbone part of the YOLOv5s network, which flexibly controls the depth of the network by controlling the number of BottleNeck modules and improves the feature extraction ability of the backbone network. The CSP2 module is introduced in the Neck part of the YOLOv5s network, which enables the information of each feature layer to be better combined together and effectively improves the Neck network’s feature fusion ability, thus enhancing the network’s characterisation ability and detection performance.

### Global context feature extraction

**Figure 3:**
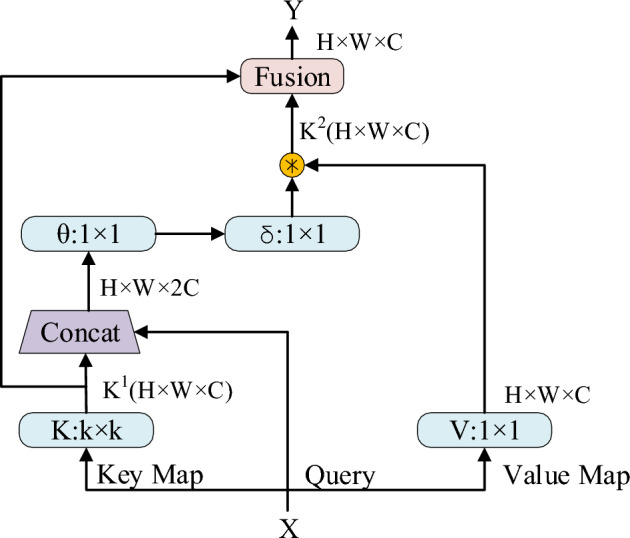
CoT structure.

YOLOv5s utilize small-sized convolutional kernels for local convolution operations to extract features. This process is effective at capturing small-scale local features from data but often struggles to capture global features. In the context of fire detection tasks, the images used are typically from outdoor scenes with more complex backgrounds and a higher level of noise interference. Small-scale local features may not adequately reflect the characteristics of fire regions and can struggle to suppress background noise. Consequently, there is a need to design network modules with the capability to extract global features, aiming to address the limitations of traditional convolutional neural networks^29^. This study draws inspiration from the recently introduced CoT module, a context-aware attention mechanism^30^. It improves traditional convolutional neural networks to enhance their global contextual learning capabilities, enabling them to excel in fire region detection tasks. Figure 3.presents the basic structure of the CoT module. Firstly, within a spatial k × k grid, the CoT module performs context encoding for all adjacent keys to obtain static context k^1^. Subsequently, it undergoes two consecutive 1 × 1 convolutions to obtain an attention matrix. Then, by multiplying this matrix with the values V of feature maps, dynamic context k^2^ is derived, which is used to capture dynamic context between inputs. Finally, it outputs a linear combination of static context k^1^ and dynamic context k^2^.

**Figure 4:**
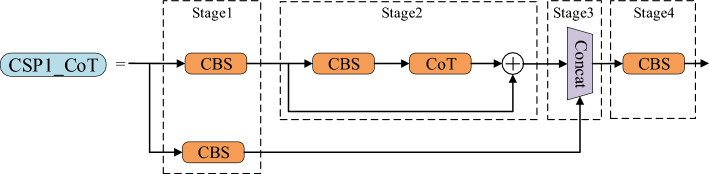
CSP1_CoT structure.

In this study, we use the CoT module to replace many BottleNeck modules in the CSP_N module to generate the CSP1_CoT module, as shown in Fig. 4. The CSP1_CoT module leverages the neighboring key mechanism to extract context information from the input image, aiding the model in better understanding the relationship between the fire and its surrounding environment. Additionally, the CoT module is capable of generating dynamic context information, which helps the model adapt to variations in the appearance of fires in different scenarios, thereby enhancing detection accuracy. Through the linear fusion of static and dynamic contexts, the CoT module provides a more comprehensive feature representation, contributing to the improvement of fire detection performance and the enhancement of model robustness. Consequently, it enables more reliable fire detection in various scenes and situations, reducing false positives and false negatives. In addition, the CSP1_CoT module reduces the number of CBS modules consisting of convolutions by reducing the number of BottleNeck modules compared to the original CSP1_N module. As a result, the CSP1_CoT module has fewer parameters, which meets the requirement of lightweight flame detection models. This improvement not only significantly boosts the performance of the fire detection model but also ensures its ability to provide reliable and accurate fire detection results in practical application. The pseudocode of the CSP1_CoT module is shown in Table 1.

**Table 1:**
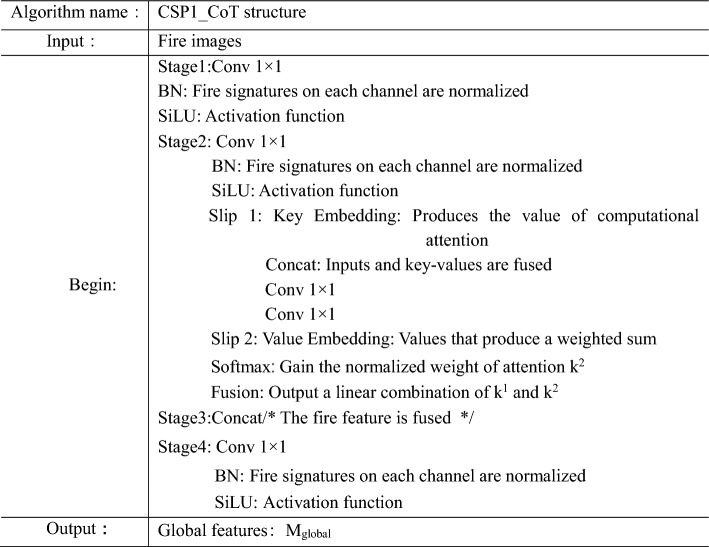
CSP1_CoT module pseudocode.

### Improvement of neck network based on SE attention mechanism

The key challenge in fire detection lies in locating fire regions with specific characteristics within complex background images, a process highly akin to the human visual attention mechanism. The attention mechanism simulates the human brain’s active perception and focus on crucial areas when processing information, imparting to machines the ability to discern data importance and thus extract more pertinent information related to the target^31^. Accordingly, this paper first introduces the current mainstream attention model, SE attention mechanism^32^, into the object detection network to improve the detection ability of the fire region. The SE attention mechanism enhances the accuracy of fire detection in two ways within the task: firstly, it dynamically adjusts the weights for each feature channel, reinforcing crucial feature channels related to fire, such as colour and shape. This, in turn, elevates the accuracy of fire detection. Secondly, the SE mechanism aids in suppressing background interference, particularly in complex backgrounds. It effectively handles variations in the appearance of fire in different contexts, such as changes in brightness, size, and the presence of smoke. Furthermore, the SE attention mechanism can improve the model performance without increasing the model parameters too much.

**Figure 5:**
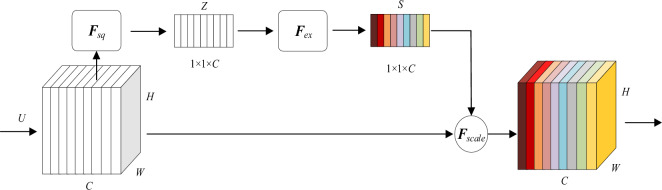
SE-Net structure.

The overall structure of the SE-Net is depicted in Fig. 5. This mechanism achieves its effects through two key steps: Squeeze and Excitation. In the Squeeze phase, *F*_*sq*_ obtains global importance weights for each channel through global average pooling, mapping the input feature maps *U* ∈ *R*^*H*×*W*×*C*^ to a vector* Z* ∈ *R*^1×1×*C*^. The process of *F*_*sq*_ is shown in Eq. (1), where *C*, *H* and *W* represent the number of channels, height and width, respectively, *u*_*c*_ ∈ *R*^*H*×*W*^ denotes the 2*D* matrix corresponding to the *c*-th channel in the feature maps *U*, *z*_*c*_ denotes a signal importance weight corresponding to *u*_*c*_ and *Z* = {*z*_1_, *z*_2_, …, *z*_*c*_, …, *z*_*C*_}.

1 $$ z_{c} = F_{sq} (u_{c} ) = \frac{1}{H \times W}\sum\limits_{i = 1}^{H} {\sum\limits_{j = 1}^{W} {u_{c} } } (i,j) $$

In the Excitation phase, *F*_*ex*_ utilizes fully connected layers to learn inter-channel relationships and obtain attention weights vector *S* ∈ *R*^1×1×*C*^. The importance weights vector *Z* firstly undergoes dimensionality reduction through a fully connected operation (${\text{W}}_{1}$) followed by activation using the ReLU function and then undergoes dimensionality expansion through another fully connected operation (${\text{W}}_{2}$). The attention weights are constrained between 0 and 1 using the Sigmoid activation function. The process is depicted in Eq. (2).

2 $$ s = F_{ex} (z_{c} ) = Sigmoid(W_{2} ({\text{Re}} LU(W_{1} z_{c} ))) $$

Finally, these attention weights are applied to the feature maps through element-wise multiplication (denoted by *F*_*scale*_), allowing the network to focus more on important features while suppressing less relevant ones, thus enhancing network performance.

**Figure 6:**
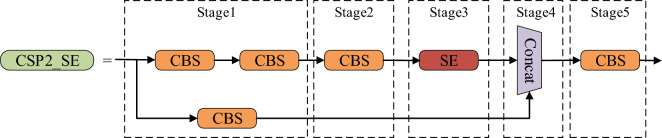
CSP2_SE structure.

Therefore, the SE module was introduced into the YOLOv5s network. Diverging from conventional module integration methods, the SE module was inserted into the first CSP2 module of the Neck network, thereby creating the CSP2_SE module, as illustrated in Fig. 6. The purpose of this approach was to further enhance the network’s feature extraction and fusion capabilities, thereby adapting the model for fire detection tasks. Fire detection typically entails dealing with dynamic, irregularly shaped, and strongly illuminated fire targets, which exhibit distinct characteristics compared to conventional object detection tasks. Hence, the insertion method of the CSP2_SE module takes into full consideration the distinctive nature of fire detection tasks. Within the context of fire detection, the CSP2_SE module assists in adjusting the weights of different-scale feature maps, facilitating improved adaptability to multi-scale targets and enhancing the robustness of the fire detection model. The subsequent experimental section will provide a comparative analysis between the traditional SE module insertion method and the CSP2_SE module in terms of their effectiveness. The pseudocode of the CSP2_SE module is shown in Table 2.

**Table 2:**
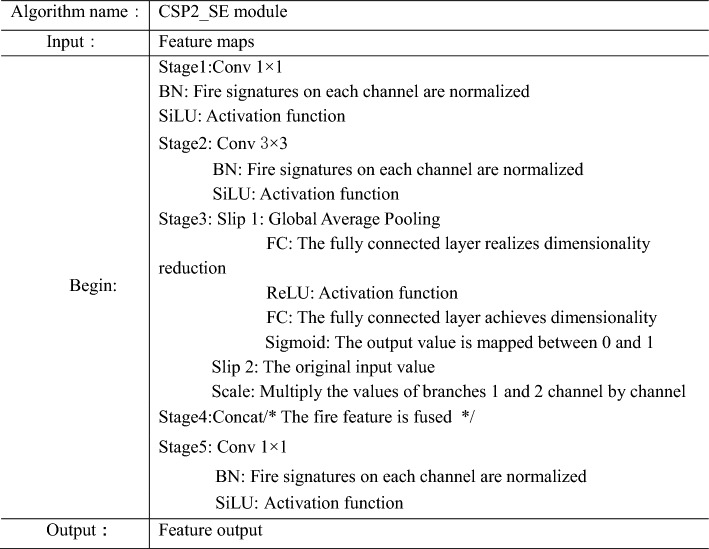
CSP2_SE module pseudocode.

### Small object detection layer

**Figure 7:**
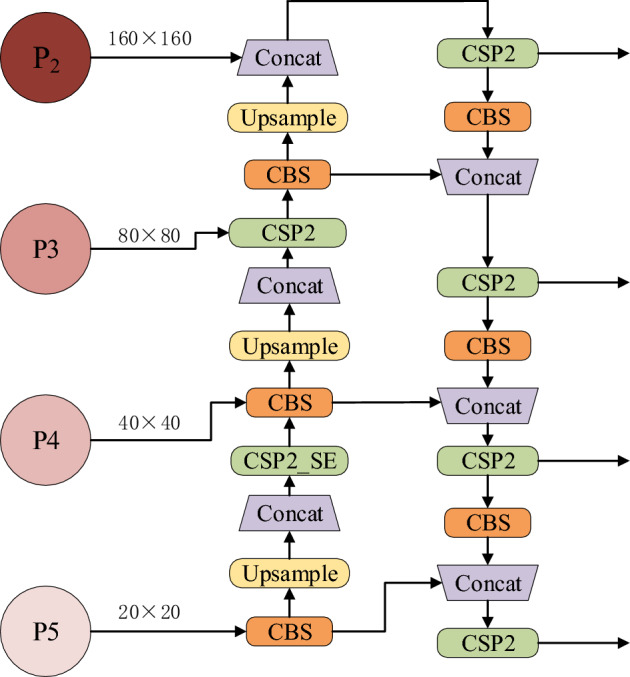
The improved Neck structure.

In real-world fire scenarios, fires exhibit significant scale variations, with a prevalence of small-scale fires. Additionally, small-scale fires often possess low contrast and minute visual features, making them prone to being overlooked or misclassified^33^. To enhance fire recognition, we have optimized the Neck module within the YOLOv5s base model. The original Neck module consists of three network layers, taking feature maps with sizes of 80 × 80, 40 × 40, and 20 × 20 as inputs. However, due to the input image resolution being 640 × 640, the feature map sizes obtained after multiple operations are significantly compressed. This results in smaller areas for small target regions (such as fires), making the targets blurrier and less conducive to fire detection tasks. To enhance the detection performance of small objects, this paper introduces an output feature map (P2) with a resolution of 160 × 160 into the CSP1_1 module of the Backbone structure, serving as the input to the Neck module. Subsequently, the output results are obtained through feature fusion and convolution operations within the original Neck module. After adding the P2 feature map, an upsampling node is introduced to the P3 feature map to fuse it with P2. The P2 feature map, with a receptive field of 4 × 4, preserves more information related to small objects, and this information can be propagated to other detection layers, enhancing the network’s feature fusion capability. The modified Neck network structure is illustrated in Fig. 7. The optimized small object fire detection layer performs better in feature extraction and target detection, significantly improving the recognition performance of small object fires. This, in turn, enhances the accuracy and reliability of fire detection.

### Focal-EIoU loss function

In YOLOv5s, the employed loss function is CIoU, which is an evolution of the DIoU loss function. It introduces a penalty parameter αv and takes into account the aspect ratio of the regression box as well as the distance between the centers of the ground truth box and the predicted box^34^. The CIoU expression is shown in Eq. (3).

3 $$ L_{CIoU} = 1 - IoU + \frac{{\rho^{2} (b,b^{gt} )}}{{c^{2} }} + \alpha \nu $$

In the formula,v is used to describe the consistency between the predicted bounding box and the target bounding box by utilizing the tangent angle. h and w represent the height and width of the predicted box, while *h(gt)* and *w(gt)* represent the height and width of the ground truth (real) box. The expressions for *v* and *α* are as shown in Eq. (4) and Eq. (5).

4 $$ \nu = \frac{4}{{\pi^{2} }}(\arctan \frac{{w^{gt} }}{{h^{gt} }} - \arctan \frac{w}{h})^{2} $$

5 $$ \alpha = \frac{\nu }{(1 - IoU) + \nu } $$

However, the CIoU loss function has a limitation in that it only considers aspect ratio as a factor. If two boxes have the same aspect ratio and their centers align with the original image, but their width and height values differ, the CIoU loss may be the same for both, even if it doesn’t align with the regression target box. Therefore, we will use the EIoU loss function instead of the original CIoU loss function. The EIoU loss function consists of three components: overlap loss, center distance loss, and width-height loss. Unlike CIoU, the width-height loss in EIoU measures the difference in width and height between the target box and the anchor box. The expression for the EIoU loss function is shown in Eq. (6)

6 $$ L_{EIoU} = 1 - IoU + \frac{{\rho^{2} (b,b^{gt} )}}{{c^{2} }} + \frac{{\rho^{2} (w,w^{gt} )}}{{C_{w}^{2} }} + \frac{{\rho^{2} (h,h^{gt} )}}{{C_{h}^{2} }} $$

where *C*_*w*_ and *C*_*h*_ respectively represent the width and height of the minimum bounding box that covers both the target box and the anchor box.

Considering the issue of training sample imbalance in bounding box regression (BBR), where high-quality anchor boxes are much less common than low-quality ones, poor-quality samples negatively impact gradients and reduce regression accuracy. To address this problem, this paper introduces the Focal loss function and combines it with the EIoU loss function to form the new Focal-EIoU loss function^35^. This loss function places more emphasis on high-quality anchor boxes, with higher-quality boxes contributing more to the loss function, thereby accelerating model convergence and improving regression accuracy. The expression for the Focal-EIoU loss function is shown in Eq. (7).

7 $$ L_{Focal - EIoU} = IoU^{\gamma } L_{EIoU} $$

where *γ *reflects the degree of suppression of outliers.

### The overall architecture of improved model

**Figure 8:**
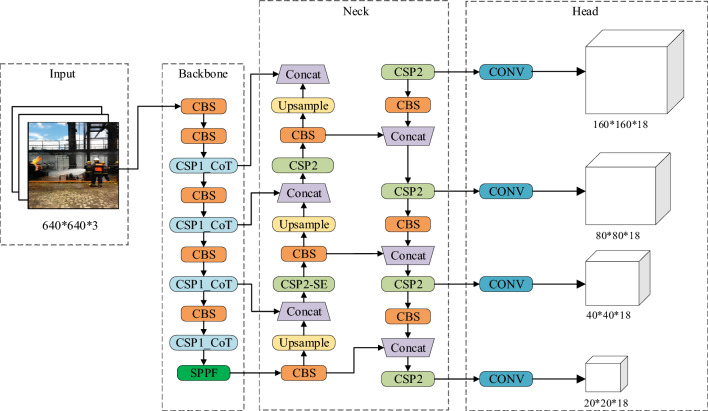
The architecture of the proposed fire detection model based on improved YOLOv5s.

Figure 8. shows the improved fire detection algorithm framework based on YOLOv5s. The algorithm is mainly divided into four parts: the input end, the backbone network, the neck network, and the output end. The input terminal dynamically adjusts the input image size to 640 × 640. The backbone network fuses the self-attention CoT module and the CSP1 module to form a new CSP1_CoT module, which not only reduces the model parameters but also enhances the feature extraction ability of the backbone network and improves the accuracy of fire detection. After extracting the fire features of the input image, the neck network uses the SE attention mechanism and the CSP2 module to form a new CSP2_SE module and dynamically adjusts the weight of the feature channel to enhance the detection ability of the target fire. At the same time, in order to improve the detection ability of small target fires, an upsampling node is added to fuse with the backbone network, and the original 3-scale detection is changed to 4-scale detection. The output of the feature map with a receptive field size of 4 × 4 and a resolution of 160 × 160 is added to enhance the detection performance of small target fires.

## Experiment and results

### Dataset construction

**Figure 9:**
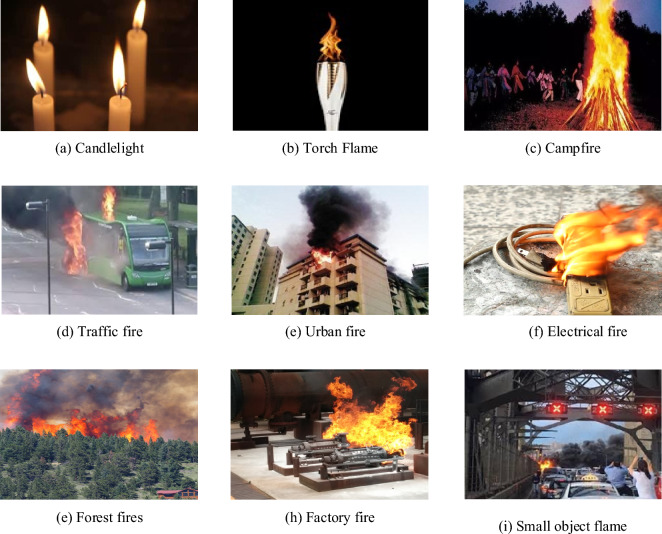
Samples from our constructed fire dataset.

In order to ensure that the model possesses strong generalization capabilities, this study collected a substantial number of fire images from various sources, including the publicly available video dataset CVPR Lab Fire^36^, the BoFire dataset^37^, the COCO dataset, and fire datasets shared by Zhang Dasheng and others^38^, as well as from the internet (e.g., Baidu and Google). More than 10,400 high-quality fire images were carefully selected and annotated using the LabelImg tool. To enhance the model’s generalization ability, the dataset includes a wide range of fire scenes, from stable and distinct fires like candles, torches, and campfires to complex and diverse fire scenarios such as forest fires, traffic fires, and urban fires. Sample images from the our constrcuted fire dataset are shown in Fig. 9.

**Figure 10:**
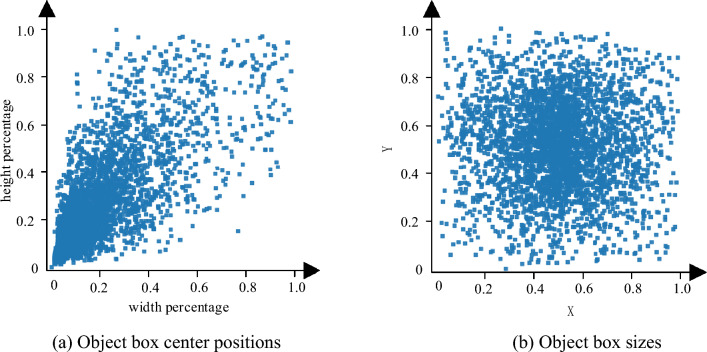
Distribution of object boxes in the dataset.

These scenes generated fires of various shapes and sizes. Finally, the dataset was randomly divided into training, validation, and test sets in a 7:2:1 ratio for model training and evaluation. To validate the dataset’s quality, the distribution of object bounding box center positions and sizes was visualized, as shown in (a) and (b) of Fig. 10.

In Fig. 10, the colour depth of the points represents the sparsity of the bounding boxes. Based on (a) and (b) in the Fig. 10, it can be observed that the distribution of bounding box positions in the dataset is relatively uniform, and the bounding boxes primarily encompass small targets. This distribution aligns well with real-world scenarios.

### Experimental platform setup and experimental design

The experimental platform for this study utilized a 64-bit Linux operating system with the following hardware specifications: CPU model—Intel(R) Xeon(R) Platinum 8255C CPU @ 2.5GHz, GPU model—NVIDIA GeForce RTX 3080. The model was implemented using the PyTorch deep learning framework, with a development environment running Python 3.8 and CUDA 11.6.

This study employed the same training parameters to train both the original YOLOv5s model and the improved version. The initial learning rate (lr0) was set to 0.01, the cyclic learning rate (lrf) to 0.02, the batch size to 16, and the number of epochs to 300. The optimizer used was Stochastic Gradient Descent (SGD). Furthermore, we also use the image flipping, Mosaic and Mixup data enhancement algorithms on the data, which makes the data more diverse and improves the generalisation ability of the model. After training, the results of the improved model were compared with the original model, and performance was also compared with mainstream object detection algorithms to validate the algorithm’s performance.

### Evaluation metrics

In this paper, the mAP, Model Parameters, and FPS were used as evaluation indicators. mAP is used to evaluate the effect of object detection and classification, and the higher the mAP value, the better the algorithm performance. Parm is the total number of parameters inside the model and is used to measure the complexity of the model. The larger the Parm, the more complex the model. FPS is the number of frames per second of the detected image. The higher the frame rate, the faster the processing speed in the actual inspection task. The calculations for these evaluation metrics are shown in Eqs. (8) to (11)

8 $$ P = \frac{TP}{{TP + FP}} $$

9 $$ R = \frac{TP}{{TP + FN}} $$

10 $$ AP = \int_{0}^{1} {P(R)dR} $$

11 $$ mAP = \frac{1}{N}\sum\limits_{{\text{i = 1}}}^{N} {AP_{i} } $$

where R is the recall rate, TP is the number of fire samples that are correctly identified, FP is the number of fire samples that are incorrectly identified, FN is the number of non-fire samples that are incorrectly identified, AP is the correct rate (P) is obtained by integrating the recall rate (R) in the interval of 0–1, and if the IoU threshold is set to 0.5, it represents the evaluation index mAP@0.5.

## Analysis of experimental results

### Validation of model improvement

**Figure 11:**
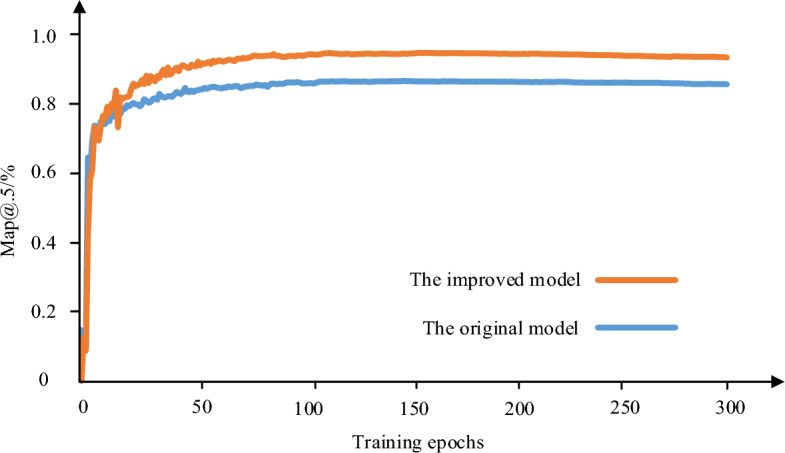
Model accuracy comparison.

To validate the performance improvement of the model, this study trained both the original model and the new model under the same experimental conditions. The mAP@0.5 was used as the performance evaluation metric for both models. A higher mAP@0.5 value indicates higher detection accuracy and better performance. The mAP@0.5 values are presented in Fig. 11. In the Fig. 11, the improved model achieves a detection accuracy of 96%, which is 8.9% higher than the original model.

In addition, this paper also validates the SE module insertion method proposed in the previous section and compares the effect of the traditional insertion position with the insertion position in this paper. In order to further validate the performance of the CSP2_SE proposed in this paper compared to other attention modules in the fire detection task, we inserted the SimAM^39,40^, CA^41^ and EMA^42^ attention modules respectively into the same position. We start training the model under the same experimental conditions, and the experimental results are shown in Table 3. As shown in Table 3, the CSP2_SE module proposed in this paper can significantly improve the accuracy and precision of the model, and the training time and the number of model parameters are also reduced accordingly.

**Table 3:** Experimental results of SE module insertion positions.

	Precision /%	mAP@0.5/%	Parm /10^6^	Time/h
Traditional SE module insertion position	84.5	85.3	6.965	7.92
SimAM	80.7	84.3	6.950	8.06
CA	79.4	84.6	7.013	7.99
EMA	81.6	84.5	7.029	8.175
Ours	94.8	96.0	6.935	7.8

In addition, this paper designed six sets of experiments to validate the impact of different improvement strategies on the YOLOv5s model, and the experimental results are shown in Table 4. In the table, ‘√’ indicates the use of the improvement strategy, while ‘ × ’ represents that the strategy was not used. As can be seen from Table 4, the CSP1_CoT module, the SE attention mechanism, and the addition of a detection layer for small targets of the three ways of introducing the CSP1_CoT module to the YOLOv5s model, the CSP1_CoT module has the greatest effect on improving the fire detection ability of the model. Introducing the CSP1_CoT module to the YOLOV5s model reduced the number of YOLOV5s model parameters while increasing the model detection accuracy by 5.1%. Then, the SE attention mechanism and the detection layer for small-target fires were gradually added to the YOLOV5s model, and the detection accuracy of the improved model was again improved by 1.7% and 1% compared with the accuracy of the YOLOV5s model with only the CSP1_CoT module introduced. Then, we changed the loss function to Focal-EIoU to further improve the model accuracy by 1%. Finally, the improved model improved the detection accuracy by 8.8% compared to the YOLOV5s model.

**Table 4:** Ablation experiments.

Model	Improvement strategies				mAP@0.5/%	Parm /10^6^
	CSP1_CoT	SE	Small Object	Focal-EIoU		
YOLOv5s	×	×	×	×	87.2	7.013
1	√	×	×	×	92.3	6.798
2	×	√	×	×	89.5	7.024
3	×	×	√	×	90.7	7.166
4	√	√	×	×	94.0	6.800
5	√	√	√	×	95.0	6.944
6	√	√	√	√	96.0	6.935

## Horizontal comparison of model performance

To validate the performance of the improved fire detection model based on the YOLOv5s model proposed in this paper, we tested SSD, Faster R-CNN, Fire-YOLO, YOLOv3-tiny, YOLOv-4tiny, YOLOv5m, YOLOv5l, YOLOv5s, YOLOv7-tiny, YOLOv8s, YOLOv9 models, as well as the fire detection models mentioned in the literature^20^, literature^33^ and literature^43^ under the same experimental conditions, and conducted comparative experiments with the fire detection model proposed in this paper. In this experiment, accuracy, detection precision, model weights, FPS and real-time detection time are used as evaluation metrics for model performance.

According to the experimental results in Table 5, the improved model based on the YOLOv5s model performs optimally in terms of precision and accuracy, and although the detection speed is slightly lower than that of the original model, it still achieves an FPS of 85 frames per second, which meets the real-time detection requirements. The improved model has a reference count of only 14.6MB, which is more suitable for deployment on resource-constrained embedded devices. Therefore, the fire detection model proposed in this paper can have high execution efficiency under the premise of guaranteeing detection accuracy, which is of some reference significance for practical applications.

**Table 5:** Comparative Experimental Results.

Model	Precision /%	mAP@0.5/%	Weight Size/MB	FPS	Time/ms
SSD	82.8	78.1	90.6	22	45.5
Faster R-CNN	83.5	87.3	315	32	31.2
YOLOv3-tiny	81.4	82.6	17.4	180	5.6
YOLOv4-tiny	49.6	79.9	23.6	188	5.3
YOLOv5m	86.2	87.4	42.1	66	15.2
YOLOv5l	86.8	87.8	88.5	64	5.6
YOLOv7-tiny	83.7	86.5	11.7	90	11.1
YOLOv5s	85.9	87.2	14.4	105	9.5
YOLOv8s	86.5	86.9	22.5	81	12.3
YOLOv9	85.3	88.8	102.8	62	16.2
Literature^20^	86.3	84.1	14.9	87	11.4
Literature^33^	82.1	78.9	16.9	66	15
Literature^43^	88.9	90.3	49.6	42	23.8
Ours	94.8	96.0	14.6	85	11.7

**Figure 12:**
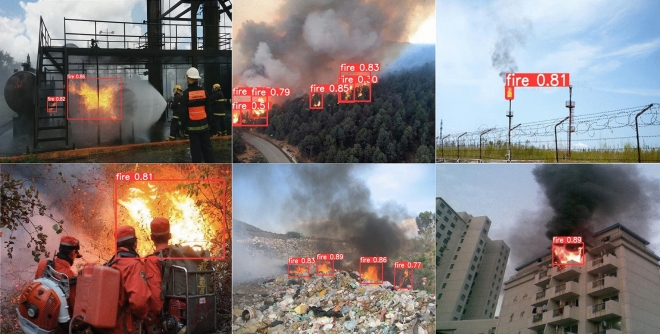
The fire image recognition results of the YOLOv5s model.

**Figure 13:**
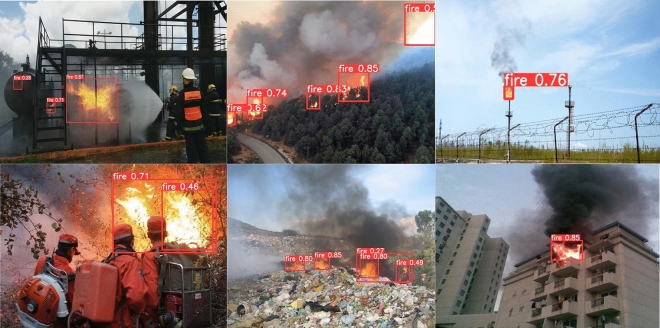
The fire image recognition results of our model.

To demonstrate that the fire detection model proposed in this paper can recognize fires in various scenarios, we selected fire images from different scenarios, including urban fires, forest fires, fires that are easily confused with the background, and small fires, and compared the results with the original model. The experimental results are shown in Figs. 12 and 13. From the detection results, it can be observed that in Fig. 12, the YOLOv5s model misidentifies the markings on the cylinder and the sunlight-illuminated clouds as fires, and its accuracy in detecting small fires is also low. The improved fire detection model can accurately identify fire targets, reduce false positives, and improve the accuracy of detecting small fires.

## Conclusions

In this paper, a fire detection algorithm based on YOLOv5s model is proposed. Firstly, the CSP1_CoT module is introduced into the backbone structure of the YOLOv5s model to reduce the model parameters and enhance the feature extraction capability of the backbone network. Then, the SE attention mechanism is introduced into the neck of the YOLOv5s model, and a small target detection layer is added to optimise the detection accuracy of the model for small targets. Finally, the loss function is changed to Focal-EIoU to further improve the detection accuracy of the model. Through experimental verification, the mAP@0.5 of the improved model reaches 96%, the precision reaches 94.8%, the weight size of the model is only 14.6MB, and the detection FPS is 85. Compared with the original model, the model proposed in this paper can be deployed in embedded devices more easily because it can improve the accuracy and precision while taking into account the model’s light weight. In the subsequent work, we should continue to expand the dataset to improve the generalisation ability of the model and adjust the network structure to achieve higher detection accuracy and faster detection speed.

## Data Availability

The datasets used during the current study are available from the corresponding author on reasonable request.

## Abbreviations

CoT: Contextual transformer: a transformer-style module
CSP1_CoT: Cross stage partial 1_contextual transformer
Focal-EIoU: Focal-efficient intersection over union: a loss function
mAP@.5: Mean average precision@.5: evaluation indicators for the model
FPS: Frames per second: evaluation indicators for the model
Faster-RCNN: Fast region-based convolutional network: target detection networks
R-FCN: Region-based fully convolutional networks: target detection networks
SSD: Single shot multibox detector: target detection networks
YOLO: You only look once: target detection networks
Bi-FPN: Bi-directional feature pyramid network: feature-fusion network
PANet: Path aggregation network: feature-fusion network
CBAM: Convolutional block attention module: an attention mechanism
CSP2_SE: Cross stage partial 2-squeeze-and-excitation
CIoU: Complete IoU: a loss function
SPPF: Spatial pyramid pooling fast: feature-fusion network
BN: Batch normalization: normalisation operation
SiLU: Sigmoid linear unit: activation function
FC: Fully connected: classification function
ReLU: Rectified linear unit: activation function
DIoU: Distance IoU: activation function
SimAM: A simple, parameter-free attention module: an attention mechanism
CA: Coordinate attention: an attention mechanism
EMA: Efficient multi-scale attention: an attention mechanism
